# Symptom prevalence in patients with advanced heart failure and its association with quality of life and activities of daily living

**DOI:** 10.1007/s11136-024-03823-9

**Published:** 2024-11-06

**Authors:** Moritz Blum, Karen McKendrick, Laura P. Gelfman, Nathan E. Goldstein

**Affiliations:** 1 Brookdale Department of Geriatrics and Palliative Medicine, Icahn School of Medicine at Mount Sinai, New York, NY USA; 2 James J. Peters Veterans Affairs Medical Center, Geriatric Research Education and Clinical Center (GRECC), Bronx, NY USA; 3 Department of Cardiovascular Surgery, Deutsches Herzzentrum der Charité Medical Heart Center of Charité and the German Heart Institute Berlin, Augustenburger Platz 1, 13353 Berlin, Germany

**Keywords:** Advanced heart failure, Symptoms, Quality of life, Activities of daily living

## Abstract

**Background:**

Quality of life (QOL) and functional status are two key outcomes for patients with advanced heart failure (HF). We examined the association of eleven symptoms with QOL and functional status impairment in patients with advanced HF.

**Methods and results:**

This was a retrospective analysis of baseline data from a multi-center, cluster-randomized controlled trial (NCT01459744) which enrolled patients with an implanted cardioverter-defibrillator and advanced HF at high-risk for mortality. Study instruments included the Condensed Memorial Symptom Assessment Scale, the Kansas City Cardiomyopathy Questionnaire QOL subscale, and the number of activities of daily living (ADL) patients had difficulties with. The study included 413 subjects. In generalized linear models which were adjusted for baseline characteristics, the total number of symptoms was significantly associated with worse QOL, as was the presence of each individual symptom, except constipation. Lack of energy demonstrated the strongest negative association with QOL. Similarly, the total number of symptoms was associated with a higher number of ADL difficulties (i.e., worse functional status). The presence of pain, lack of energy and drowsiness was individually associated with more ADL difficulties.

**Conclusion:**

Among patients with advanced HF, a higher number of symptoms and specific individual symptoms were associated with worse QOL and functional status.

**Supplementary Information:**

The online version contains supplementary material available at 10.1007/s11136-024-03823-9.

## Lay summary

People living with advanced heart failure typically experience shortness of breath and leg swelling. We found that many of them also experience additional unpleasant symptoms e.g., lack of energy, difficulty sleeping and dry mouth.

The more symptoms patients with heart failure experience, the worse is their quality of life and the more help they need with their daily activities (e.g. bathing, dressing, toileting).

Some symptoms seem to be more harmful than others. The symptoms which had a negative effect on both quality of life and daily activities were lack of energy, pain and drowsiness.

**Supplementary Information:**

The online version contains supplementary material available at 10.1007/s11136-024-03823-9.

## Introduction

Heart failure (HF) is highly prevalent and despite major therapeutic advances, prognosis remains poor [1, 2]. Even though disease trajectories may vary, most patients eventually progress to a state of refractory symptoms despite optimal therapy, which is referred to as advanced HF [3].

Patients with advanced HF experience a severe burden of symptoms. Per definition, patients with HF suffer from the typical, congestion-related signs and symptoms of HF, including breathlessness, orthopnea, reduced exercise tolerance, fatigue and lower extremity swelling [4]. Previous studies have also documented a high prevalence of less typical symptoms, such as difficulty sleeping, dry mouth, drowsiness and pain [5–8].

How symptoms impact quality of life (QOL) and activities of daily living (ADL) among patients with advanced HF remains unclear. Maintaining QOL and improving functional status are of paramount importance for patients with HF [9, 10]. Previous studies established a significant association between the total number of symptoms and worse QOL in patients with advanced HF [11, 12]. Yet, which individual symptoms are most detrimental to QOL remains unclear. Similarly, the association of both, the overall number of symptoms and individual symptoms, with functional status impairment has never been comprehensively studied. These associations are fundamentally important, as a better understanding of the relationship between symptoms and both QOL and functional status can help clinicians design clinical interventions to ameliorate suffering and improve outcomes in this population.

Therefore, in this study, we sought to examine how the total number of symptoms and the presence of individual symptoms correlate with QOL and functional status impairment in a large population of patients with advanced HF.

## Methods

This study was a retrospective analysis of data from the multi-center, single-blind, cluster-randomized controlled Working to Improve diScussions about DefibrillatOr Management (WISDOM) trial, which was conducted at six academic medical centers in the United States and tested if an educational intervention for clinicians and automatic reminders could increase discussions about deactivation of implantable cardioverter defibrillator (ICD) devices in patients with advanced HF (NCT01459744). Study design and primary outcomes are described in detail elsewhere [13, 14]. The study was approved by the Program for the Protection of Human Subjects at the Icahn School of Medicine at Mount Sinai and institutional review boards at each of the participating sites.

### Study population

The WISDOM trial enrolled adult HF patients with an ICD and a high likelihood of dying according to prespecified criteria [13]. Both inpatients and outpatients were eligible for inclusion, if they had experienced repeated HF hospitalizations in the last 12 months, New York Heart Association (NYHA) Class III or IV HF, and evidence of end-organ failure, such as blood urea nitrogen > 43 mg/dL, serum creatinine > 2.75 mg/dL, systolic blood pressure < 115 mmHg, or if they were older than 70 years of age (for more details see Supplementary Table 1). For this retrospective analysis, participants with incomplete data on the Condensed Memorial Assessment Scale (CMSAS) were excluded. Details on excluded patients can be found in Supplementary Table 2.

### Study measurements

At baseline, all study participants were interviewed to obtain self-reported demographic data. Trained research coordinators abstracted comorbidities, NYHA Class, left ventricular ejection fraction (LVEF), candidate status for ventricular assist device (VAD) or heart transplantation, current medication, laboratory values and from electronic medical records at baseline at the trial sites. Baseline interviews also included the following instruments:

- *Condensed Memorial Assessment Scale* (CMSAS): This abbreviated version of the 32-item Memorial Symptom Assessment Scale – Short Form (MSAS-SF) surveys the presence of 14 common symptoms during the last week and, if present, prompts participants to rate how much they were bothered by the symptom, with good internal consistency (Cronbach’s alpha 0.85) [15]. In this study, only the physical subscale of the CMSAS which focuses on eleven physical symptoms (constipation, difficulty concentrating, difficulty sleeping, drowsiness, dry mouth, lack of appetite, lack of energy, nausea, pain, shortness of breath, weight loss) was administered, because emotional symptoms were measured with separate dedicated scales. As our research aim was to study the impact of symptom presence on quality of life and functional status, we coded the presence of individual CMSAS symptoms as binary variables (present vs. not present) irrespective of how much patients reported they were bothered by the symptom. The *total number of symptoms* refers to the number of symptoms reported, with a range of zero to eleven.
- *Kansas City Cardiomyopathy Questionnaire - Quality of Life Subscale (KCCQ-QOL)*: The 23-item KCCQ contains a three item QOL subscale which elicits patients’ self-reported enjoyment, life satisfaction, and feeling of discouragement [16]. The reliability and validity of this subscale have been demonstrated in different clinical settings (Cronbach’s alpha 0.72–0.85) [17, 18]. In our analysis investigating the interplay of symptom prevalence and QOL, we only utilized the KCCQ-QOL subscale as construct of QOL, because the KCCQ total score already includes measures of symptom prevalence. The KCCQ-QOL score can take on values from zero to 100 with higher values indicating better QOL.
- *Activities of Daily Living (ADL)*: ADL are a widely used measure of functional status and its reliability and validity are well established [19]. Patients were asked to rate the difficulty they had with ADL (dressing, bathing, eating, toileting, transferring from bed, walking). The *number of ADL difficulties* refers to the number of ADL for which patient reported some impairment, i.e., which they self-rated as, “some difficulty”, “quite a lot difficulty”, or “cannot do”. The *number of ADL difficulties* can take on values from zero to six.

### Study outcomes

The outcomes of interest for this study were, in a non-hierarchical order: the prevalence of each individual symptom, the total number of symptoms per patient, and the association of the total number of symptoms and the presence of individual symptoms with the KCCQ-QOL score and the number of ADL difficulties.

### Statistical analysis

Population characteristics and baseline variables are presented as number (percentage) or mean ± standard deviation, as appropriate. Independent variables for univariable analysis were the total number of symptoms and the presence of each individual symptom. To study the impact of symptoms on QOL and functional status, we constructed separate generalized linear models (GLM) using the KCCQ-QOL score and the number of ADL difficulties, respectively, as the dependent variable. We performed an unadjusted and an adjusted analysis. Based on their clinically evident association with both QOL and functional status, we decided a priori to include the following co-variables for adjustment: age, gender, the number of comorbidities, chronic kidney disease, LVEF, NYHA class. In addition, the multivariable model with KCCQ-QOL score included the number of ADL difficulties as a covariable, and vice versa. For each GLM, we report coefficients and 95% confidence intervals (95% CI) in parentheses and p-values. For this study, we defined a significance level of < 0.05. For GLMs with the KCCQ-QOL score as response variables, the coefficients can be interpreted as unit change of the KCCQ-QOL score if the respective symptom is present. For GLMs with the number of ADL difficulties as response variables, the coefficients could be interpreted as unit change of the number of ADL difficulties if the respective symptom is present. The statistical analysis was performed using Stata Statistical Software: Release 16 (StataCorp LLC, College Station, TX, United States).

## Results

### Population characteristics

The final study population comprised 413 patients. Population characteristics are detailed in Table 1. Overall, the mean age was 62.1 ± 13.5 years, 69.3% identified as male and 49.6% identified as Non-Hispanic White, 37.0% identified as Non-Hispanic Black, 13.5% identified as Hispanic, and 2.0% identified as Asian. The mean left ventricular ejection fraction (LVEF) was 24.6 ± 9.9% and most patients were in NYHA Class III (77.5%) or Class IV (12.7%). Nearly half of patients (46.7%) had ischemic HF. Overall, 40.8% and 37.4% were considered candidates for ventricular assist device (VAD) and heart transplantation, respectively. The most frequent comorbid conditions were coronary artery disease (56.6%), previous myocardial infarction (43.4%), diabetes mellitus (45.0%), and chronic kidney disease (31.3%). Most patients were treated with a diuretic (92.7%), a beta-blocker (89.1%), and angiotensin-converting enzyme inhibitor (44.4%) or an angiotensin receptor blocker (18.5%). About half of the patients (53.4%) received an aldosterone antagonist (53.4%) and 10.9% received inotrope therapy.

**Table 1:** Population characteristics

	*N* = 413
Age – years, mean ± SD	62.1 ± 13.5
Gender, male – no. (%)	286 (69.3)
Race/Ethnicity – no. (%)	
Asian	8 (2.0)
Hispanic	55 (13.5)
Non-Hispanic Black	146 (37.0
Non-Hispanic White	196 (49.6)
College degree– no. (%)	190 (46.1)
Married/ partner – no. (%)	226 (55.3)
Comorbidities – no. (%)*	
History of myocardial infarction	162 (43.4)
Coronary artery disease	214 (56.6)
Diabetes	176 (45.0)
Malignancy	60 (15.4)
Chronic kidney disease	122 (31.3)
No. of comorbidities – mean ± SD	3.1 ± 1.8
HF Medication – no. (%)	
Beta Blocker	367 (89.1)
ACE inhibitor	183 (44.4)
Angiotensin receptor blocker	76 (18.5)
Aldosterone antagonist	220 (53.4)
Diuretic	383 (92.7)
LVEF – %	24.6 ± 9.9
Ischemic HF etiology – no. (%)	191 (46.7)
NYHA class – no. (%)	
I/II	40 (9.8)
III	317 (77.5)
IV	52 (12.7)
VAD candidate – no. (%)	168 (40.8)
Heart transplant candidate – no. (%)	154 (37.4)
No. of ADL difficulties – mean ± SD	1.5 ± 1.8
KCCQ Overall Summary Score – mean ± SD	41.4 ± 22.6
KCCQ QOL Domain Score – mean ± SD	42.6 ± 26.90
One-Year Mortality – no. (%)	66 (16.0)

Malignancy included lymphoma, leukemia and solid cancers. Abbreviations: ACEI, angiotensin converting enzyme; ADL, Activities of daily living; LVEF, left ventricular ejection fraction; NYHA, New York Heart Association; QOL, quality of life; SD, standard deviation; VAD, ventricular assist device

### Total number of symptoms and prevalence of individual symptoms

**Fig. 1:**
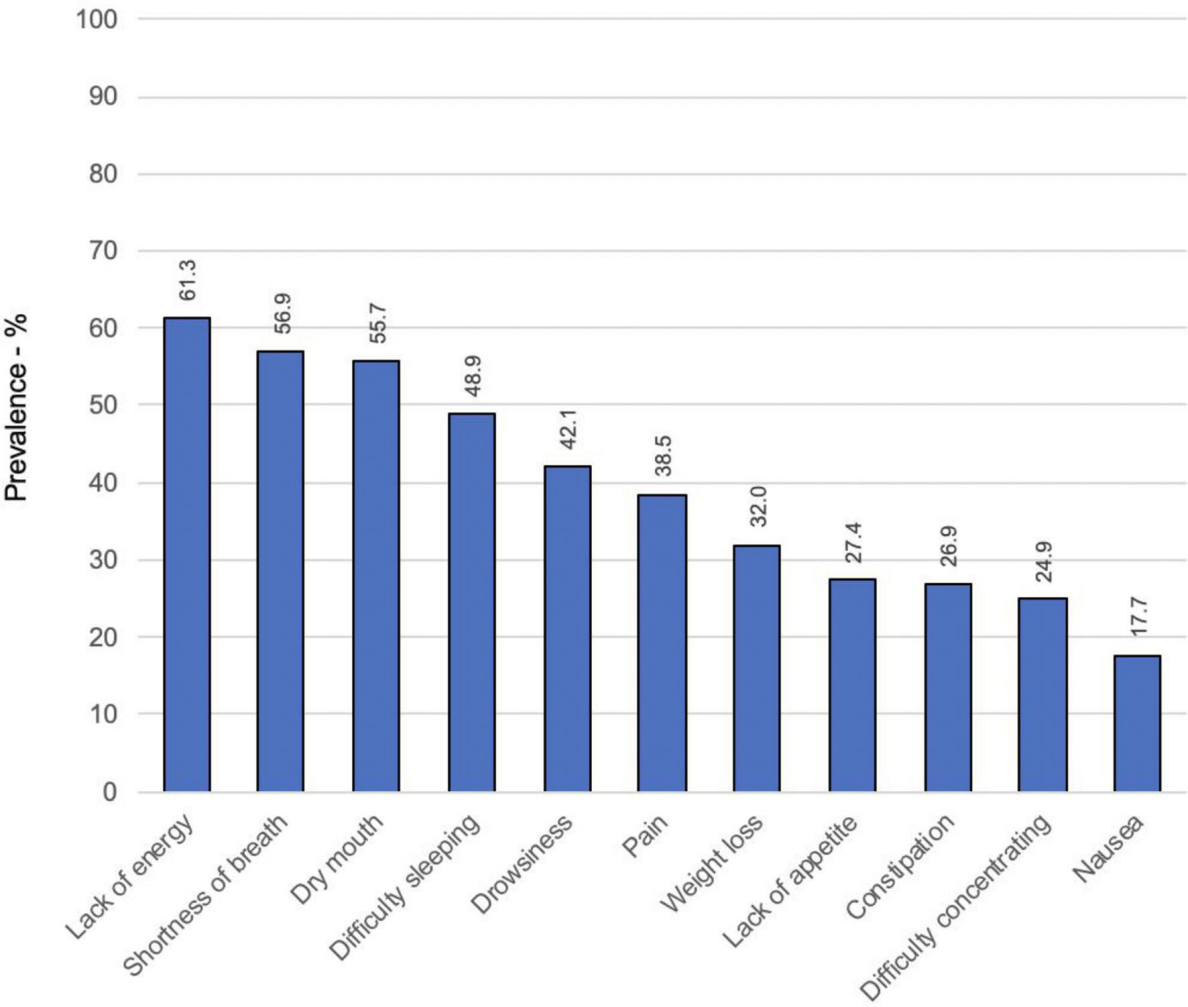
Prevalence of symptoms according to the condensed memorial assessment scale

On average, patients reported 4.3 ± 2.7 symptoms. The prevalence of individual symptoms is illustrated Fig. 1. The most prevalent symptoms, affecting more than half of the patients, were lack of energy (61.3%), shortness of breath (56.9%), and dry mouth (55.7%). Less prevalent symptoms were difficulty sleeping (48.9%), drowsiness (42.1%), pain (38.5), and weight loss (32.0%). The least prevalent symptoms were lack of appetite (27.4%), constipation (26.9%), difficulty concentrating (24.9%), and nausea (17.7%).

### Association of symptom presence and quality of life

Using GLMs, we studied the association of the total number of symptoms and KCCQ-QOL scores. A greater total number of symptoms was significantly associated with a lower KCCQ-QOL score indicating worse QOL, both in adjusted and unadjusted analyses (Table 2). Each additional symptom was associated with a -5.1 (95% CI -5.9, -4.3) reduction of the KCCQ-QOL score in the unadjusted analysis, and with a -4.1 (95% CI -5.1, -3.1) reduction of the KCCQ-QOL score in the adjusted analysis.

**Table 2:** Association of total number of symptoms and individual symptoms with the KCCQ Quality of life subscale

	Unadjusted		Adjusted	
	Coefficients (95% CI)	*p*-value	Coefficients (95% CI)	*p*-value
Number of CMSAS symptoms	-5.1 (-5.9, -4.3)	**< 0.001**	-4.1 (-5.1, -3.1)	**< 0.001**
Pain	-14.2 (-19.3, -9.1)	**< 0.001**	-7.8 (-13.2, -2.3)	**0.005**
Lack of energy	-21.5 (-26.4, -16.7)	**< 0.001**	-17.3 (-22.4, -12.2)	**< 0.001**
Lack of appetite	-20.5 (-25.9, -15.1)	**< 0.001**	-14.2 (-20.0, -8.4)	**< 0.001**
Nausea	-19.3 (-25.8, -12.9)	**< 0.001**	-9.7 (-16.9, -2.5)	**0.009**
Dry mouth	-10.2 (-15.3, -5.1)	**< 0.001**	-5.7 (-11.0, -0.4)	**0.034**
Drowsiness	-16.5 (-21.5, -11.6)	**< 0.001**	-9.5 (-14.7, -4.3)	**< 0.001**
Shortness of breath	-15.7 (-20.7, -10.8)	**< 0.001**	-9.5 (-14.7, -4.3)	**< 0.001**
Constipation	-6.0 (11.7, -0.3)	**0.04**	-0.25 (-6.1, 5.6)	0.93
Difficulty sleeping	-18.4 (-23.2, -13.6)	**< 0.001**	-10.9 (-16.1, -5.8)	**< 0.001**
Difficulty concentrating	-21.2 (-26.9, -15.6)	**< 0.001**	-13.3 (-19.3, -7.2)	**< 0.001**
Weight loss	-10.5 (-15.9, -5.1)	**< 0.001**	-5.5 (-11.0, -0.2)	**0.044**

The reported results were computed using a generalized linear model. Covariables for adjustment analysis were age, gender, the number of comorbidities, chronic kidney disease, left ventricular ejection fraction, New York Heart Association class, and the number of impaired activities of daily living

*Abbreviations* CI, confidence interval; CMSAS, Condensed Memorial Assessment Scale; KCCQ, Kansas City Cardiomyopathy Questionnaire

**Fig. 2:**
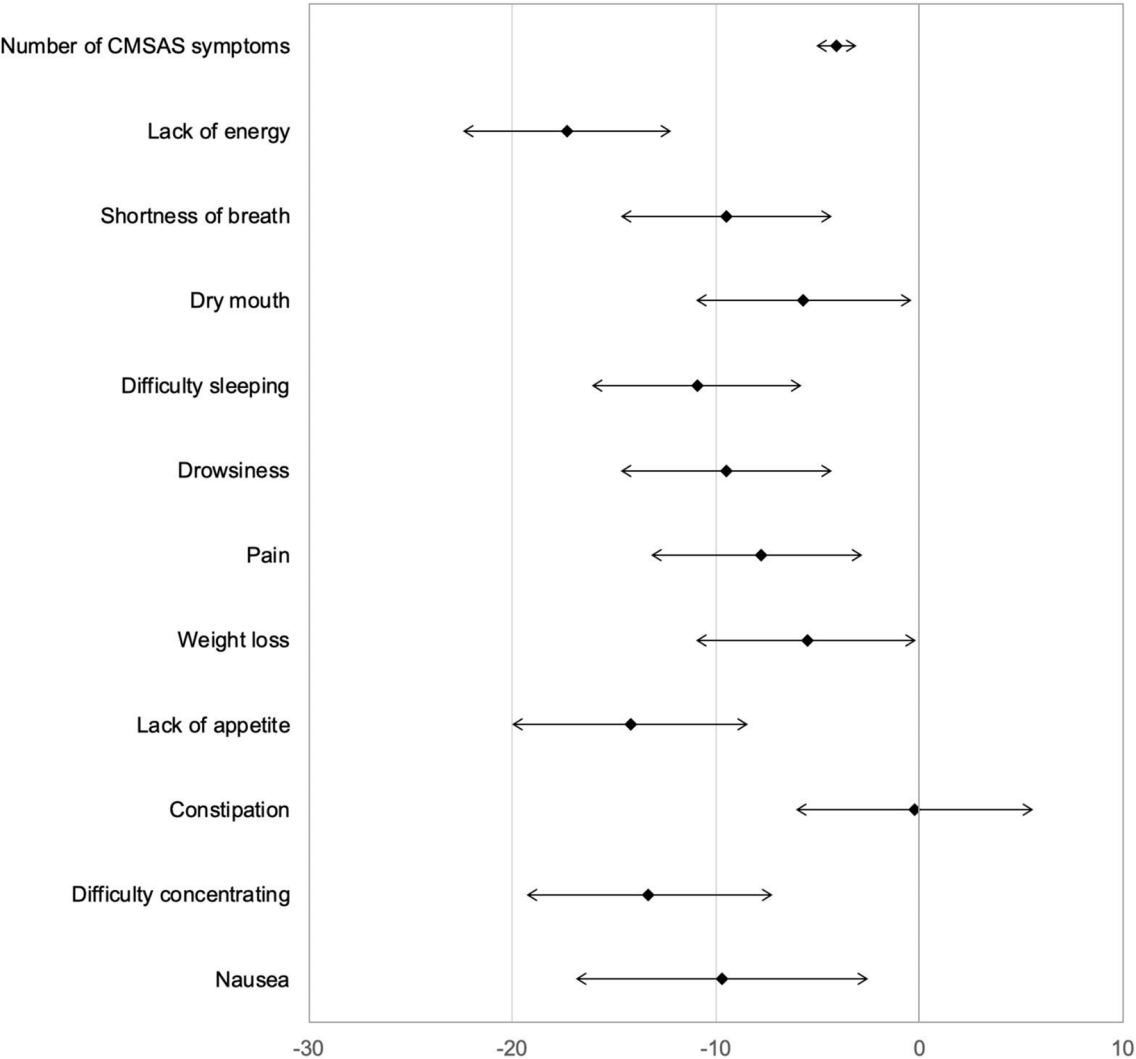
Association of symptom presence and the KCCQ quality of life subscale. *Legend* Reported are coefficients and 95% confidence intervals derived from adjusted generalized linear models. Covariates for adjustment were age, gender, the number of comorbidities, chronic kidney disease, left ventricular ejection fraction, New York Heart Association class, and the number of impaired activities of daily living. Abbreviations: CMSAS, Condensed Memorial Assessment Scale; KCCQ, Kansas City Cardiomyopathy Questionnaire

We also studied the association of the presence of each individual symptom and QOL. In the unadjusted analysis, all symptoms n were significantly associated with lower KCCQ-QOL score. After adjusting for baseline characteristics, these findings remained largely unchanged (Table 2). The strength of the association varied significantly between different symptoms (Fig. 2). Lack of energy (-17.3 [95% CI -22.4, -12.2]) was associated with the largest reduction of QOL, whereas weight loss (-5.5 [95% CI -11.0, -0.2]) and dry mouth (-5.7 [95% CI -11.0, -0.4]) were associated with the smallest reduction in QOL, in adjusted analyses. Constipation was not associated with QOL in the adjusted analysis.

### Association of symptom presence and activities of daily living

In a separate GLM, we studied the association of symptoms and the number of ADL difficulties. A higher total number of symptoms was significantly associated with more difficulties with ADL, both in adjusted and unadjusted analysis (Table 3). On average, each additional symptom increased the number of ADL difficulties by 0.21 (95% CI 0.16, 0.25) in the unadjusted analysis, and by 0.15 (95% CI 0.07, 0.22) in the adjusted analysis.

**Table 3:** Association of total number of symptom and individual symptoms with the number of activities of daily living difficulties

	Unadjusted		Adjusted	
	Coefficients (95% CI)	*p*-value	Coefficients (95% CI)	*p*-value
Number of CMSAS symptoms	0.21(0.16, 0.27)	**< 0.001**	0.15 (0.07, 0.22)	< 0.001
Pain	0.60 (0.3, 0.86)	**< 0.001**	0.39 (0.09, 0.69)	**0.01**
Lack of energy	0.80 (0.53, 1.06)	**< 0.001**	0.41 (0.07, 0.76)	**0.02**
Lack of appetite	0.74 (0.46, 1.02)	**< 0.001**	0.33 (-0.01, 0.67)	0.06
Nausea	0.62 (0.30, 0.94)	**< 0.001**	0.37 (-0.04, 0.78)	0.08
Dry mouth	0.56 (0.31, 0.80)	**< 0.001**	0.15 (-0.16, 0.46)	0.35
Drowsiness	0.72 (0.47, 0.97)	**< 0.001**	0.33 (0.03, 0.64)	**0.03**
Shortness of breath	0.58 (0.33, 0.83)	**< 0.001**	0.17 (-0.14, 0.47)	0.29
Constipation	0.35 (0.08, 0.63)	**0.012**	0.21 (-0.11, 0.54)	0.20
Difficulty sleeping	0.57 (0.32, 0.83)	**< 0.001**	0.28 (-0.01, 0.58)	0.06
Difficulty concentrating	0.59 (0.30, 0.88)	**< 0.001**	0.25 (-0.10, 0.61)	0.15
Weight loss	0.41 (0.15, 0.68)	**0.002**	0.3 (-0.00, 0.62)	0.05

Coefficients and *p*-values were derived from a generalized linear model. Covariates for adjustment analysis were age, gender, the number of comorbidities, chronic kidney disease, left ventricular ejection fraction, New York Heart Association class, and the Kansas City Cardiomyopathy Questionnaire quality-of-life subscale

*Abbreviations* CMSAS, Condensed Memorial Assessment Scale

**Fig. 3:**
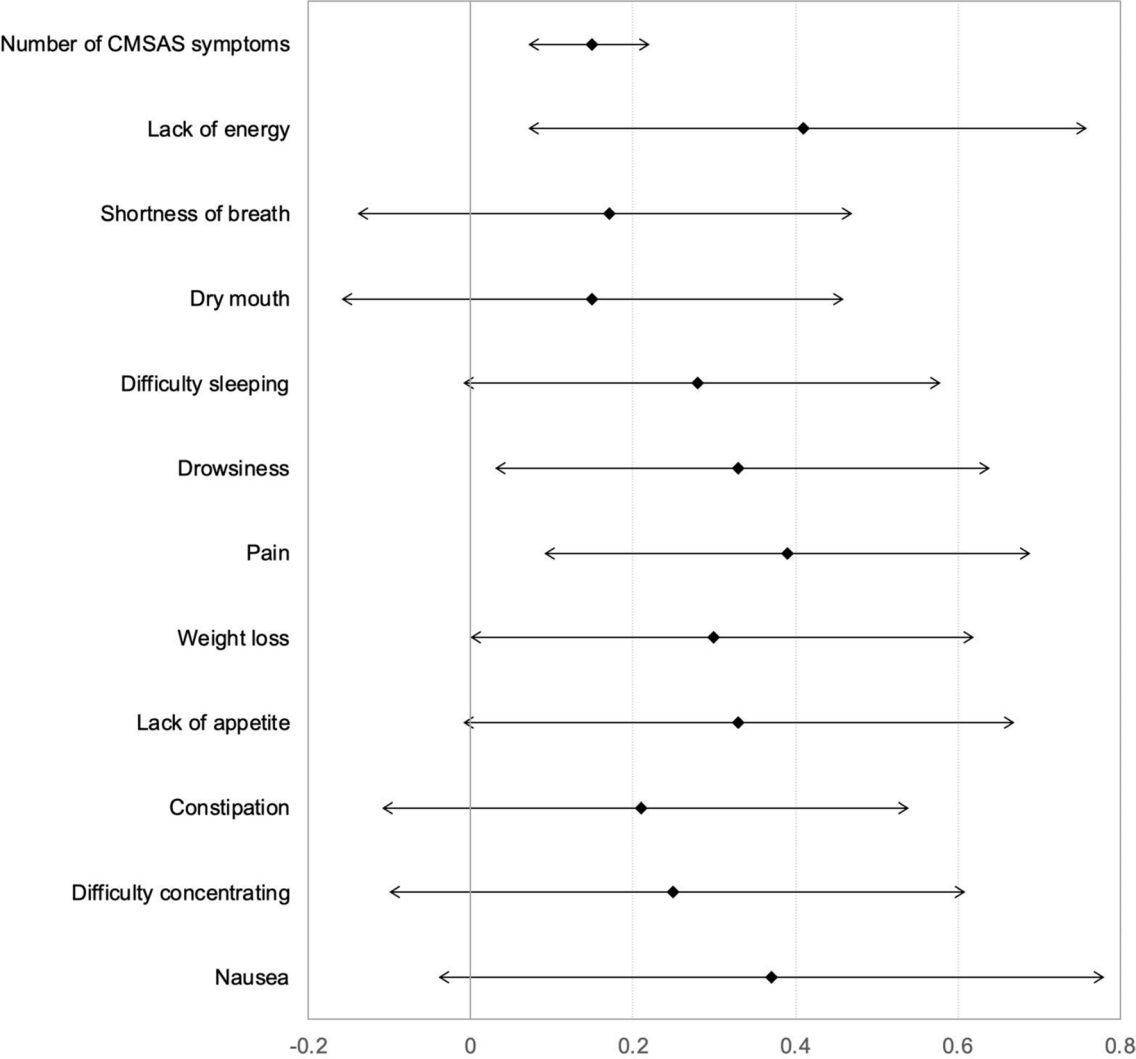
Association of symptom presence and the number of impaired activities of daily living. *Legend* Reported are coefficients and 95% confidence intervals derived from adjusted generalized linear models. Covariates for adjustment were age, gender, the number of comorbidities, chronic kidney disease left ventricular ejection fraction, New York Heart Association class, and the KCCQ quality-of-life subscale. Abbreviations: CMSAS, Condensed Memorial Assessment Scale; KCCQ, Kansas City Cardiomyopathy Questionnaire

In our examination of individual symptoms, all symptoms were significantly associated with a higher number of ADL difficulties in unadjusted analyses (Table 3). After adjustment for baseline characteristics, however, only lack of energy [0.41 (95% CI 0.07, 0.76)], pain [0.39 (95% CI 0.09, 0.69)] and drowsiness [0.33 (95% CI 0.03, 0.64)] remained significantly associated with a higher number of ADL difficulties. In addition, we found trends regarding the association of lack of appetite (0.33 [95% CI -0.01, 0.67]), nausea (0.37 [95% CI-0.04, 0.78]), difficulty sleeping (0.28 [95% CI -0.01, 0.58]) and weight loss (0.3 [95% CI -0.00, 0.62]) with a higher number ADL difficulties, but these trends were not statistically significant (Fig. 3).

### Discussion

In this retrospective cross-sectional study of patients with advanced HF, we made the following findings. First, the total number of symptoms was significantly associated with both QOL and functional status impairment. Second, the strength of association with QOL and functional status impairment differed between individual symptoms. The symptoms with both a strong association with worse QOL and functional status impairment were lack of energy, pain and drowsiness.

These findings are in line with previous studies documenting the adverse impact of symptom burden on QOL in advanced HF populations. While multiple previous studies investigated symptom prevalence in the general HF population [5–8], literature on symptom prevalence in the advanced HF sub-population remains scarce and accordingly, review articles and position papers on advanced HF rarely discuss symptoms other than those typically associated with congestion [3, 20, 21]. Two small studies documented a high symptom prevalence in this population, but one of them could not establish any associations between individual symptoms and the KCCQ-QOL score [12], and the other one found that only lack of energy, drowsiness and feeling irritable were associated with poorer quality of life [11].

Our study adds new evidence about the association of individual symptoms and QOL and highlights the importance of specific symptoms. Using a significantly larger sample than extant studies, we were able to identify negative associations of multiple individual symptoms and QOL. Specifically, the symptoms difficulty sleeping, difficulty concentrating, and lack of appetite are not routinely thought of as critical to QOL in patients with advanced HF, yet each of them was associated with at least a 10-point reduction on the KCCQ-QOL score. Of note, lack of energy was the symptom with the most detrimental impact on QOL, which was associated with a reduction of nearly 20-point reduction on the KCCQ-QOL score. Considering that KCCQ-QOL differences greater than ± 5 are generally regarded as clinically meaningful in trials, the associations we found are not just statistically significant, but rather indicative of drastic and tangible changes in health-related QOL [16].

In addition, we document the impact of individual symptoms on functional impairment, measured by the number of ADL difficulties. A small previous study using a clustering approach documented worse functional status in one symptom cluster, which also included fatigue [22]. In unadjusted analyses, we found significant associations with functional impairment for every individual symptom studied. After adjustment for baseline characteristics however, many of those associations lost significance, indicating that some symptoms were markers, rather than independent predictors, of functional status impairment. However, after adjustment, lack of energy, pain and drowsiness remained significantly associated with functional status impairment.

Our findings suggest that clinicians and researchers should direct more attention to underrecognized symptoms when developing strategies to improve QOL and functional status of patients with advanced HF. When discussing symptom assessment and symptom management, clinical cardiology textbooks and international HF guidelines focus predominantly on the classic congestion-related signs and symptoms of HF, i.e., breathlessness, orthopnea, exercise intolerance, and ankle swelling, and fatigue [4, 23]. Along the same lines, the most widely used patient-reported HF-specific outcome measure, the KCCQ, only contains items asking shortness of breath, ankle swelling, and fatigue, and does not include other highly prevalent symptoms such as difficulty sleeping or pain [16]. Our findings indicate that these symptoms are both highly prevalent, and both cumulatively and individually associated with QOL and functional impairment among patients with advanced HF. Consequently, underrecognized symptoms including pain, lack of energy, drowsiness and difficulty sleeping deserve more consideration, both by clinicians who care for patients with advanced HF on a daily basis, and by those designing targeted interventions to improve QOL and functional status for patients with advanced HF. Such interventions to better address symptom burden in the future could for instance include standardized symptom assessment using validated tools, manualized prescription of medications for symptom control, education about symptom self-management, physical and respiratory therapy, and counseling with a focus on symptom perception, processing and coping.

Limitations and strengths of the present study should be considered when interpreting our findings. First, this was a cross sectional analysis, so causal relationships between the presence of symptoms and outcomes cannot be inferred based on the associations we found. Second, this was a secondary analysis and the lack of significant associations between some symptoms and outcomes could represent a lack of statistical power to detect statistically significant differences. However, to our knowledge this is the largest systematic study of prevalence and impact of symptoms in advanced HF, and the associations of the total number of symptoms and individual symptoms remained statistically significant after adjusting for relevant baseline characteristics. Of note, we exclusively focused on physical symptoms in this analysis. Patients with advanced HF also experience burdensome emotional symptoms, namely depression and anxiety, which may also impact the perception of physical symptoms [6]. Therefore, clinicians should also consider the burden of emotional symptoms, and further research is needed to untangle the intricate relationship of physical and emotional symptoms in this population. Besides, we only studied the presence of symptoms and its associations with outcomes of interest – analyzing symptom severity instead or in addition to presence of symptoms might have led to different results. Lastly, the presence of symptoms, which were not captured in the CMSAS might have confounded this analysis.

In conclusion, in a population of patients with advanced HF, we found that a higher total number of symptoms and specific individual symptoms were associated with poorer QOL and worse functional status impairment. More awareness about the detrimental impact of under-recognized symptoms like lack of energy, pain, and difficulty sleeping could be instrumental in improving QOL and functional status for patients with advanced HF.

## Electronic supplementary material

Below is the link to the electronic supplementary material.

Supplementary Material 1

## Data Availability

Data availability requests can be addressed to the corresponding author.

## Abbreviations

ADL: Activities of daily living
CMSAS: Condensed Memorial Assessment Scale
GLM: Generalized linear model
HF: Heart failure
ICD: Implantable cardioverter-defibrillator
KCCQ: Kansas City Cardiomyopathy Questionnaire
LVEF: Left ventricular ejection fraction
MSAS-SF: Memorial Symptom Assessment Scale – Short Form
NYHA: New York Heart Association
QOL: Quality of life
VAD: Ventricular assist device
WISDOM: Working to Improve diScussions about DefibrillatOr Management
