# Supplementary material for: Symptom prevalence in patients with advanced heart failure and its association with quality of life and activities of daily living
Source: Qual Life Res. 2024 Nov 6;34(2):485–93. doi: 10.1007/s11136-024-03823-9 (PMC12135210; doi:10.1007/s11136-024-03823-9)
Supplement: Supplementary file 1 — Supplementary Material 1 [file 11136_2024_3823_MOESM1_ESM.docx]

**Supplementary Tables**

| **Supplementary Table 1. Final Inclusion Criteria of the WISDOM trial** | |
| --- | --- |
| **Inclusion Criteria** | |
| **For outpatients** | Has had 2 or more HF-related admissions in the last year  *or*  Has NYHA Class IV HF in the outpatient setting  *or*  Has NYHA Class III HF in outpatient setting  *and*  One HF-related admission in the last year  *or*  Has NYHA Class III HF in outpatient setting  *and*  Two of the following four conditions:   - Age > 70 years - Blood urea nitrogen (BUN)> 43 mg/dL - Creatinine > 2.75 mg/dL - Systolic Blood Pressure (SBP) < 115 mmHg |
| **For inpatients** | Has had at least one other HF admission in the last year  *or*  Meets all three of the following criteria at time of admission:   - BUN > 43 mg/dL - SBP <115 mmHg - Creatinine >2.75 mg/dL   *and*  Will be followed in the outpatient cardiology/HF practice with an enrolled clinician |
| **Exclusion Criteria** | |
| - Age< 18 - No active ICD (i.e., has an ICD that was not deactivated before study enrollment) - Unable to speak English or Spanish - Not having consistent and reliable access to a phone - Having a VAD at the time of enrollment | |
| More details can be found at clinicaltrials.gov (NCT01459744). Abbreviations: BUN, blood urea nitogen; HF, heart failure; ICD, implantable cardioverter-defibrilator; NYHA, New York Heart Association; WISDOM, Working to Improve diScussions about DefibrillatOr Management, VAD, ventricular assist device. | |

| **Supplementary Table 2. Sample bias analysis – Baseline characteristics for patients with complete vs. incomplete data** | | | |
| --- | --- | --- | --- |
|  | **Excluded**  **(N=150)** | **Included**  **(N=413)** | **p-value** |
| Age – years, mean ± SD | 60.9 ±14.7 | 62.1 ± 13.5 | 0.40 |
| Gender, male – no. (%) | 109 (73.7) | 286 (69.3) | 0.31 |
| Race/Ethnicity – no. (%) |  |  |  |
| Non-Hispanic White | 54 (38.6) | 196 (49.6) | 0.02 |
| Non-Hispanic Black | 61 (43.6) | 146 (37.0) | 0.17 |
| Hispanic | 26 (17.8) | 55 (13.5) | 0.20 |
| Asian | 3 (2.1) | 8 (2.0) | 0.93 |
| College degree– no. (%) | 70 (48.6) | 190 (46.1) | 0.61 |
| Married/ partner – no. (%) | 81(54.7) | 226 (55.3) | 0.91 |
| Comorbidities – no. (%) |  |  |  |
| History of MI | 40 (31.8) | 162 (43.4) | 0.02 |
| Coronary artery disease | 66 (52.0) | 214 (56.6) | 0.36 |
| Diabetes | 32 (41.6) | 176 (45.0) | 0.58 |
| Malignancy | 7 (9.1) | 60 (15.4) | 0.15 |
| Chronic kidney disease | 19 (25.0) | 122 (31.3) | 0.28 |
| No. of comorbidities – mean ± SD | 2.33 ± 1.7 | 3.06 ± 1.8 | 0.001 |
| HF Medication – no. (%) |  |  |  |
| Beta Blocker | 121 (83.5) | 367 (89.1) | 0.08 |
| ACEI | 46 (31.7) | 183 (44.4) | 0.008 |
| ARB | 34 (23.5) | 76 (18.5) | 0.19 |
| Aldosterone antagonist | 72 (49.7) | 220 (53.4) | 0.44 |
| Diuretic | 136 (90.7) | 383 (92.7) | 0.42 |
| LVEF – %, mean ± SD | 23.30 ± 10.8 | 24.58 ± 9.9 | 0.21 |
| Ischemic HF etiology – no. (%) | 51 (38.1) | 191 (46.7) | 0.08 |
| NYHA class – no. (%) |  |  |  |
| I/II | 6 (4.4) | 40 (9.8) | 0.05 |
| III | 105 (77.2) | 317 (77.5) | 0.94 |
| IV | 25 (18.4) | 52 (12.7) | 0.10 |
| VAD candidate – no. (%) | 63 (43.8) | 168 (40.8) | 0.53 |
| Heart Tx candidate – no. (%) | 62 (43.1) | 154 (37.4) | 0.23 |
| No. of ADL difficulties – mean ± SD | 2.0 ± 1.2 | 1.5 ± 1.8 | 0.25 |
| KCCQ Overall Summary Score – mean ± SD | 48.9 ± 24.8 | 41.4 ± 22.6 | 0.14 |
| KCCQ QOL Domain Score – mean ± SD | 51.5 ± 25.4 | 42.6 ± 26.0 | 0.17 |
| One-Year Mortality – no. (%) | 17 (11.3) | 66 (16.0) | 0.17 |
| Malignancy included lymphoma, leukemia and solid cancers. Abbreviations: ACEI, angiotensin converting enzyme; ADL, Activities of daily living; HF, heart failure; KCCQ, Kansas City Cardiomyopathy Questionnaire; LVEF, left ventricular ejection fraction; MI, myocardial infarction; NYHA, New York Heart Association; QOL, quality of life; SD, standard deviation; VAD, ventricular assist device. | | | |
